# Maresin-1 and maresin-2 as synovial fluid biomarkers in psoriatic, rheumatoid, and osteoarthritis

**DOI:** 10.3389/fmed.2026.1856412

**Published:** 2026-07-14

**Authors:** Sukru Demir, Bugra Can, Omer Esmez, Mustafa Ata Aydin, Sevval Yagmur Gencer, Furkan Bildirici, Süleyman Serdar Koca, İbrahim Sahin, Suleyman Aydin

**Affiliations:** 1 Department of Orthopedics and Traumatology, Medical School, Firat University, Elazig, Türkiye; 2 Clinical of Science, School of Medicine, Gazi University, Ankara, Türkiye; 3 Department of Medical Biochemistry and Clinical Biochemistry, (Firat Hormones Research Group), Medical School, Firat University, Elazig, Türkiye; 4 Department of İnternal Medicine (Rheumatology), Medical School, Firat University, Elazig, Türkiye; 5 Department of Medical Biology, Medical School, Erzincan Binali Yildirim University, Erzincan, Türkiye

**Keywords:** arthritis, biomarker, osteoarthritis, psoriatic arthritis, rheumatoid arthritis, synovial fluid

## Abstract

**Objective:**

To investigate synovial fluid levels of maresin-1 (MaR-1) and maresin-2 (MaR-2) in patients with psoriatic arthritis (PsA), rheumatoid arthritis (RA), osteoarthritis (OA), and controls, and to assess their ability to differentiate PsA from RA.

**Methods:**

This prospective cross-sectional study included 88 patients undergoing knee arthrocentesis for joint effusion between September 2025 and February 2026. RA was classified according to the 2010 ACR/EULAR criteria, PsA using CASPAR criteria, and OA based on clinical and radiographic assessment. Synovial fluid MaR-1 and MaR-2 concentrations were measured by enzyme-linked immunosorbent assay (ELISA) and expressed as ng/mL. Group comparisons were performed using parametric or non-parametric statistical tests as appropriate. Receiver operating characteristic (ROC) curve analysis evaluated the diagnostic performance of MaR-1 and MaR-2 for distinguishing PsA from RA, including area under the curve (AUC), sensitivity, specificity, and optimal cut-off values. Logistic regression analysis assessed the association between MaR-1 levels and PsA.

**Results:**

Significant intergroup differences were observed for age, visual analogue scale (VAS) scores, and synovial MaR-1 concentrations (all *p* < 0.01). MaR-1 levels were significantly higher in PsA than RA (*p* < 0.01), whereas MaR-2 showed more modest differences among groups (*p* < 0.05). ROC analysis demonstrated good discriminatory performance of MaR-1 for differentiating PsA from RA, with an AUC of 0.815 (95% CI, 0.675–0.933). A cut-off value of 3.252 ng/mL provided 68.2% sensitivity and 86.4% specificity. Higher MaR-1 levels were significantly associated with PsA in unadjusted logistic regression analysis (OR = 1.62, 95% CI 1.18–2.22; *p* = 0.003). MaR-1 and MaR-2 levels were moderately positively correlated (Spearman’s *r* = 0.620, *p* < 0.001).

**Conclusion:**

Synovial MaR-1 concentrations were markedly elevated in PsA compared with RA and demonstrated good diagnostic discrimination, supporting the potential role of pro-resolving mediators as biomarkers in inflammatory arthropathies. The clinical significance of MaR-2 appears more limited and requires validation in larger studies.

## 1 Introduction

Inflammation is a tightly regulated biological process essential for host defense and tissue repair; however, failure of resolution mechanisms can lead to persistent inflammation, progressive tissue damage, and chronic disease (1). Emerging evidence indicates that chronic inflammatory conditions are not solely driven by excessive activation of pro-inflammatory pathways but also by impaired endogenous programs responsible for terminating inflammation and restoring tissue homeostasis (2, 3). This paradigm shift has prompted increasing interest in molecular mediators that actively orchestrate the resolution phase of inflammation.

Rheumatoid arthritis (RA) is a chronic inflammatory arthropathy characterized by synovial hyperplasia and the expansion of activated fibroblast-like synoviocytes (FLS), which play a central role in disease pathogenesis (4). These cells promote persistent inflammation through the production of pro-inflammatory cytokines and matrix-degrading enzymes, including metalloproteinases, thereby contributing to cartilage destruction and progressive joint damage. Although RA and psoriatic arthritis (PsA) share several clinical manifestations, they differ considerably in their underlying immunopathological mechanisms (5). RA is primarily driven by adaptive immune activation, autoantibody formation, and cytokines such as tumor necrosis factor-*α* (TNF-α) and interleukin-6 (IL-6) (6). In contrast, PsA is more strongly linked to the IL-23/IL-17 axis, entheseal inflammation, and a greater contribution of innate immune pathways (7, 8). Osteoarthritis (OA), once considered purely degenerative, is now recognized as a heterogeneous disorder involving low-grade inflammation and dysregulated resolution pathways (9). These disease-specific mechanisms likely generate distinct lipid mediator signatures within the synovial microenvironment (10). Despite extensive investigation of metalloproteinases as diagnostic biomarkers for differentiating inflammatory arthropathies, their clinical applicability remains limited, highlighting the need for more specific and mechanistically relevant biomarkers (11, 12).

In this context, omega-3 polyunsaturated fatty acids have gained considerable attention as precursors of specialized pro-resolving mediators (SPMs), a class of bioactive lipid molecules that actively regulate the resolution of inflammation without inducing immunosuppression. Among these, maresin-1 (MaR-1) and maresin-2 (MaR-2), biosynthesized from docosahexaenoic acid by macrophages, have emerged as key regulators of inflammatory homeostasis (13–16). MaR-1 has been shown to limit neutrophil infiltration, promote neutrophil apoptosis, and enhance macrophage-mediated efferocytosis, thereby facilitating the clearance of inflammatory debris (17). Furthermore, it induces macrophage polarization toward an anti-inflammatory M2 phenotype and suppresses the production of pivotal pro-inflammatory cytokines such as tumor necrosis factor-*α* and interleukin-1β (18). Beyond innate immunity, MaR-1 also modulates adaptive immune responses by suppressing pathogenic T helper cell subsets, including Th1 and Th17, while promoting regulatory (19, 20).

MaR-2, another member of the maresin family, further highlights the complexity of pro-resolving lipid mediator networks, although its biological functions remain less extensively characterized (21, 22). Together, MaR-1 and MaR-2 represent a coordinated system that contributes to the active resolution of inflammation (23, 24). Within the joint microenvironment, particularly in the synovium, MaR-1 and MaR-2 are thought to modulate FLS activity by suppressing the production of inflammatory cytokines and metalloproteinases, thereby limiting cartilage degradation and tissue damage. Synovial fluid, which directly reflects intra-articular biochemical and cellular processes, provides a valuable medium for identifying disease-specific biomarkers (25). Previous lipid mediator profiling studies have demonstrated the presence of MaR-1 in RA synovial fluid and suggest its involvement in regulating key inflammatory signaling pathways, including PI3K/Akt and NF-κB (26). However, comparative data evaluating synovial levels of MaR-1 and MaR-2 across different arthropathies—such as psoriatic arthritis (PsA) and osteoarthritis (OA)—remain scarce.

Therefore, the present study aimed to investigate synovial fluid levels of MaR-1 and MaR-2 in patients with RA, PsA, OA, and healthy controls, and to evaluate their potential relevance as biomarkers of intra-articular inflammatory activity as well as their utility in distinguishing RA from other joint diseases.

## 2 Materials and methods

This study was conducted as a prospective, observational, cross-sectional investigation. Between September 1, 2025, and February 1, 2026, consecutive patients presenting with knee joint effusion and undergoing arthrocentesis for diagnostic or therapeutic purposes at the Orthopedics and Traumatology Clinic of Fırat University Hospital were enrolled.

The study population included patients with RA, PsA, and OA, as well as a control group of medically healthy individuals who underwent diagnostic or therapeutic knee arthrocentesis for non-inflammatory mechanical conditions and had no clinical, laboratory, or radiographic evidence of inflammatory arthritis, autoimmune disease, or active infection.

All participants were recruited consecutively to minimize selection bias. The study protocol was reviewed and approved by the Fırat University Clinical Research Ethics Committee (approval no. 28.08.2025–38,327), and the study was conducted in accordance with the principles of the Declaration of Helsinki. Written informed consent was obtained from all participants prior to inclusion. Eligible participants were adults aged ≥18 years with clinically and/or radiologically confirmed knee joint effusion and a clear clinical indication for arthrocentesis.

The diagnosis of RA was confirmed according to the 2010 ACR/EULAR classification criteria (27), while PsA was established using the CASPAR criteria (28). OA was diagnosed on the basis of clinical assessment (29) in conjunction with knee radiographs, with disease severity graded according to the Kellgren–Lawrence classification (30).

Patients with suspected or confirmed septic arthritis, evidence of crystal-induced arthritis (gout or calcium pyrophosphate deposition disease) in the aspirated joint, or a recent history of joint infection were excluded from the study. In addition, individuals with a history of knee trauma or surgery within the preceding year, those receiving active antibiotic therapy, patients with concomitant systemic inflammatory diseases (e.g., systemic lupus erythematosus or vasculitis), and cases in whom clinical judgment suggested that such conditions could substantially confound the results were not included. Samples with insufficient volume or with marked hemolysis or contamination were also excluded from the final analysis. To minimize the potential influence of intra-articular treatments, patients who had received corticosteroid, hyaluronic acid, or platelet-rich plasma injections into the aspirated knee within the previous 12 weeks were excluded.

During arthrocentesis, demographic and clinical data were systematically recorded, including age, sex, smoking status, and relevant comorbidities. Knee pain severity was assessed at the same visit using a visual analogue scale (VAS) to reflect clinical symptom burden (31). In the OA group, radiographic disease severity was determined on the basis of available knee radiographs and graded according to the Kellgren–Lawrence classification.

Knee synovial fluid was aspirated using standard sterile arthrocentesis techniques and collected into sterile tubes. When clinically indicated, a portion of the sample was allocated for routine synovial fluid analyses, including cell count with differential, Gram staining and culture, and crystal examination. The aliquots reserved for MaR-1 and MaR-2 measurements were processed promptly following sample collection. To remove cellular components and debris, the samples were centrifuged under appropriate conditions. The resulting supernatant was then divided into suitable volumes and stored at −80 °C until analysis, thereby avoiding repeated freeze–thaw cycles.

Synovial fluid concentrations of MaR-1 and MaR-2 in the knee were quantified using commercially available, human-specific enzyme-linked immunosorbent assay (ELISA) kits in accordance with the manufacturers’ instructions (SunRed Biotechnology, China; Maresin-1 Catalog No. 201–12-7349, Maresin-2 Catalog No. 201–12-7396). Measurements were performed based on a double-antibody sandwich ELISA principle.

For MaR-1, the assay detection range was 0.05–10 ng/mL with an analytical sensitivity of 0.042 ng/mL, while for MaR-2 the detection range was 0.05–15 ng/mL with an analytical sensitivity of 0.047 ng/mL. The reported intra-assay and inter-assay coefficients of variation were <10 and <12%, respectively. Absorbance values were read using a reader ChroMate P4300 (Awareness Technology Instruments, United States)., and washing steps were carried out with a Bio-Tek ELX50 microplate washer. Concentrations were expressed in ng/mL. Laboratory personnel performing the assays were blinded to the clinical group assignments. Synovial fluid Maresin-1 and -2 measurements were tested for experimental validity according to previously described methods, and it was reported that the kits used measured with the same sensitivity as in medical serum samples (32).

### 2.1 Statistical analyses

Statistical analyses were carried out using IBM SPSS Statistics version 26.0 (IBM Corp., Armonk, NY, United States). The distributional characteristics of continuous variables were examined visually with histograms and Q–Q plots. Continuous data are reported as mean ± standard deviation or median with interquartile range, as appropriate, while categorical variables are expressed as counts and percentages.

For group comparisons, one-way analysis of variance (ANOVA) was applied to age and VAS scores, whereas categorical variables, including smoking status, were analyzed using the chi-square test. Differences in MaR-1 and MaR-2 levels among the four study groups were assessed using the Kruskal–Wallis test. The ability of MaR-1 and MaR-2 to discriminate between PsA and RA was evaluated by receiver operating characteristic (ROC) curve analysis, with calculation of the area under the curve (AUC) and corresponding 95% confidence intervals; the optimal cut-off point was defined according to the Youden index. The relationship between MaR-1 and MaR-2 concentrations was explored using Spearman correlation analysis. All analyses were two-tailed, and statistical significance was set at a *p* value of less than 0.05.

## 3 Results

**Figure 1:**
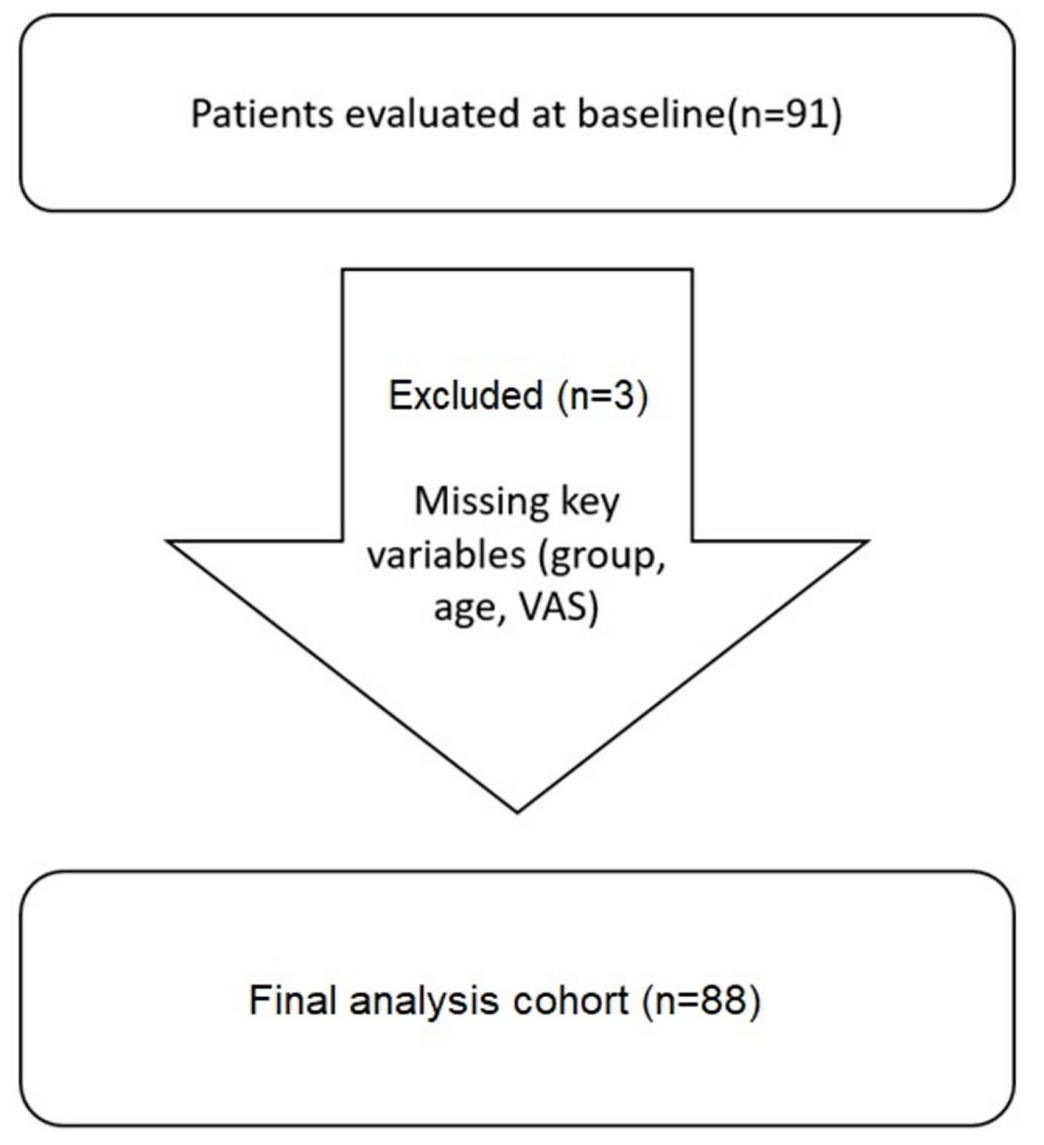
Participant flow diagram illustrating screening, exclusion reasons, and formation of the final analytical cohort. VAS, visual analogue scale.

A total of 88 participants were included in the analysis, comprising four groups of equal size (*n* = 22 each): PsA, RA, OA, and controls. A balanced design with equal group sizes was intentionally adopted to optimize statistical power and minimize potential bias related to unequal sampling. The participant flow diagram summarizes eligibility assessment, exclusion criteria, and the derivation of the final analytical cohort (Figure 1).

Baseline demographic and clinical characteristics are presented in Table 1. The mean age of the overall cohort was 45.0 ± 18.1 years, with a significant difference observed across groups (*p* < 0.01); patients in the OA group were older on average than those in the other groups. VAS pain scores also differed significantly between groups (*p* < 0.01), with the highest mean VAS recorded in the OA group (6.5 ± 1.4) and the lowest in the control group (2.1 ± 1.8). Overall, 42 of 88 participants (47.7%) were current smokers, and no significant difference in smoking prevalence was detected among the groups (*p* = 0.178; Table 1).

**Table 1:** Baseline demographic and clinical characteristics by study group (age, VAS score, and smoking status).

Group	*n*	Age (mean ± SD)	VAS score (mean ± SD)	Smoking, *n* (%)
Control	22	35.8 ± 15.1	2.1 ± 1.8	7 (31.8)
RA	22	39.1 ± 17.8	5.4 ± 0.8	13 (59.1)
OA	22	64.5 ± 9.7 ^**a,b,c**^	6.5 ± 1.4 ^**a,b,c**^	9 (40.9)
PsA	22	40.4 ± 13.4	4.3 ± 0.8	13 (59.1)
Total	88	45.0 ± 18.1	4.6 ± 2.1	42 (47.7)

*One-way ANOVA was used for age and VAS scores; the chi-square test was used for smoking status. OA, osteoarthritis; PsA, psoriatic arthritis; RA, rheumatoid arthritis; SD, standard deviation; VAS, visual analogue scale. ^a^OA group vs Control group (*p* < 0.01); ^b^OA group vs RA group (*p* < 0.01); ^c^OA group vs PsA group (*p* < 0.01).

**Figure 2:**
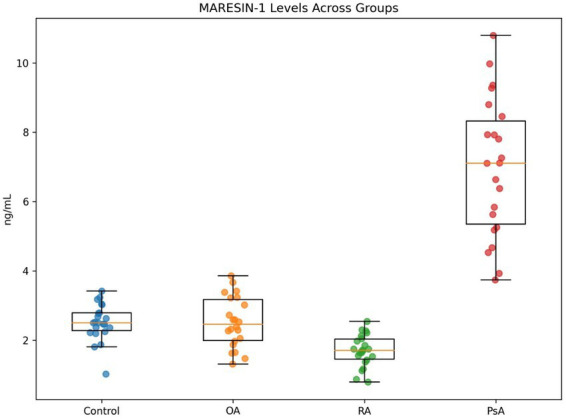
Distribution of synovial fluid MaR-1 levels in PsA, RA, OA, and control groups (box-and-whisker plots with individual data points). MaR-1, maresin-1; OA, osteoarthritis; PsA, psoriatic arthritis; RA, rheumatoid arthritis.

**Figure 3:**
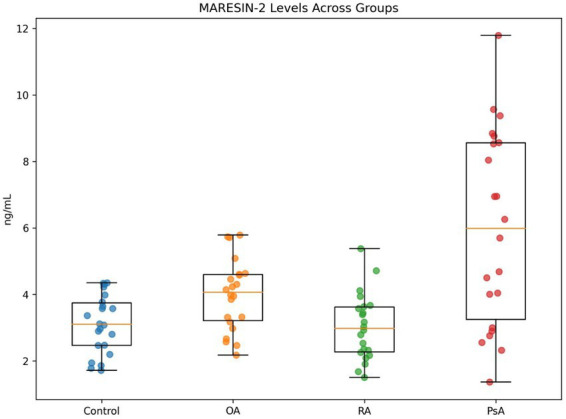
Distribution of synovial fluid MaR-2 levels in PsA, RA, OA, and control groups (box-and-whisker plots with individual data points). MaR-2, maresin-2; OA, osteoarthritis; PsA, psoriatic arthritis; RA, rheumatoid arthritis.

The distribution of MaR-1 and MaR-2 concentrations according to study groups is summarized in Table 2. MaR-1 levels differed significantly among the groups (*p* < 0.01), with the highest values observed in the PsA group. MaR-2 concentrations also showed a statistically significant between-group difference, although to a lesser extent (*p* < 0.05; Table 2). Graphical representations of group-wise distributions of MaR-1 and MaR-2 are presented in Figures 2, 3, respectively.

**Table 2:** Distribution of synovial fluid MaR-1 and MaR-2 levels across study groups.

Group	*n*	MaR-1 ng/mL (mean ± SD)	MaR-2 ng/mL (mean ± SD)
Control	22	2.50 ± 0.77	2.70 ± 1.62
RA	22	2.13 ± 1.65	4.21 ± 3.34
OA	22	2.76 ± 1.39	4.22 ± 2.66
PsA	22	6.01 ± 3.72 ^a,b,c^	7.69 ± 6.45 ^d,e,f^

*Between-group comparisons were performed using the Kruskal–Wallis test due to non-normal distribution and upper-limit values. MaR-1, maresin-1; MaR-2, maresin-2; OA, osteoarthritis; PsA, psoriatic arthritis; RA, rheumatoid arthritis; SD, standard deviation. ^a^PsA group vs Control group (*p* < 0.01); ^b^PsA group vs RA group (*p* < 0.01); ^c^PsA group vs OA group (*p* < 0.01). ^d^PsA group vs Control group (*p* < 0.05); ^e^PsA group vs RA group (*p* < 0.05); ^f^PsA group vs OA group (*p* < 0.05).

The magnitude of the difference between the PsA and RA groups was examined in greater detail. Mean MaR-1 levels were substantially higher in patients with PsA (6.01 ± 3.72 ng/mL) compared with those with RA (2.13 ± 1.65 ng/mL; Table 2). The mean between-group difference was 3.88 ng/mL, with a 95% confidence interval of 2.10–5.65 ng/mL (*p* < 0.01). The corresponding effect size was large, as indicated by a Hedges’ g value of 1.32.

**Figure 4:**
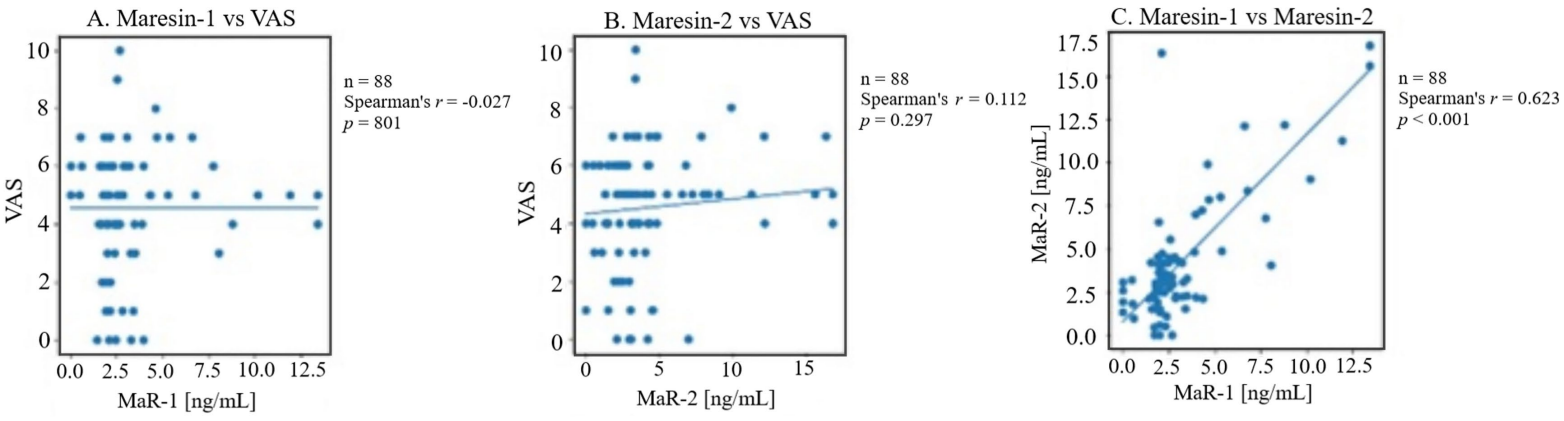
Correlation analyses between maresins and pain intensity. MaR-1, maresin-1; MaR-2, maresin-2; VAS, visual analogue scale.

Correlation analysis revealed a significant, moderate-to-strong positive association between MaR-1 and MaR-2 levels across the entire cohort (Spearman’s *r* = 0.620, *p* < 0.001). This relationship was particularly pronounced in the PsA group (*r* = 0.933, *p* < 0.001), whereas a weaker, moderate correlation was observed in the RA group (*r* = 0.485, *p* = 0.022; Figure 4).

A significant positive correlation was found between MaR-1 and MaR-2 levels. (Spearman *r* = 0.623, *p* < 0.001). No significant relationship was observed between MaR-1 levels and VAS scores. (*r* = −0.027, *p* = 0.801). Similarly, no significant correlation was found between MaR-2 and VAS scores. (*r* = 0.112, *p* = 0.297).

**Figure 5:**
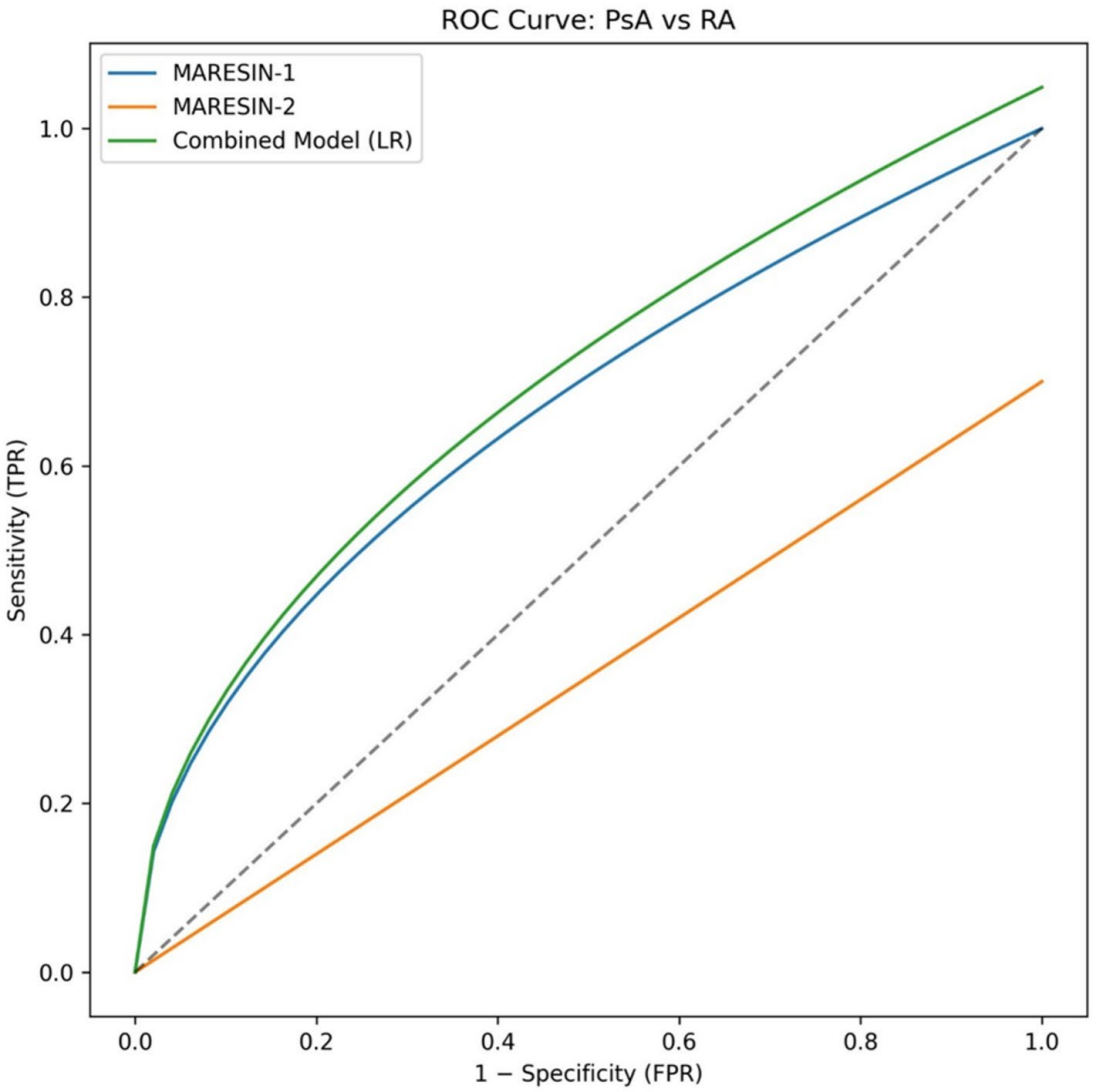
ROC curves for MaR-1, MaR-2, and the combined model in differentiating PsA from RA. MaR-1, maresin-1; MaR-2, maresin-2; PsA, psoriatic arthritis; RA, rheumatoid arthritis; ROC, receiver operating characteristic.

Receiver operating characteristic (ROC) analysis was performed to assess the ability of MaR-1 and MaR-2 to discriminate between PsA and RA. MaR-1 demonstrated good discriminatory performance, with an AUC of 0.815 (95% CI, 0.675–0.933; Figure 5; Table 3). At the optimal cut-off value of 3.252 ng/mL defined by the Youden index, MaR-1 yielded a sensitivity of 68.2% and a specificity of 86.4%. In contrast, the standalone discriminative capacity of MaR-2 was limited (AUC 0.597; 95% CI, 0.410–0.781; Table 3).

**Table 3:** ROC/AUC analysis for discrimination between PsA and RA (AUC, 95% confidence interval, cut-off value, sensitivity, and specificity).

Parametres	AUC (95% CI)	Cut-off (Youden)	Sensitivity	Specificity
MaR-1	0.815 (0.675–0.933)	3.252	0.682	0.864
MaR-2	0.597 (0.410–0.781)	9.036	0.409	0.955
Combined (MaR-1 + MaR-2)	0.864 (0.740–0.959)	0.367*	0.955	0.682

*Cut-off in the combined model represents the predicted probability threshold derived from the logistic regression model (Youden index). AUC, area under the curve; MaR-1, maresin-1; MaR-2, maresin-2; ROC, receiver operating characteristic.

**Figure 6:**
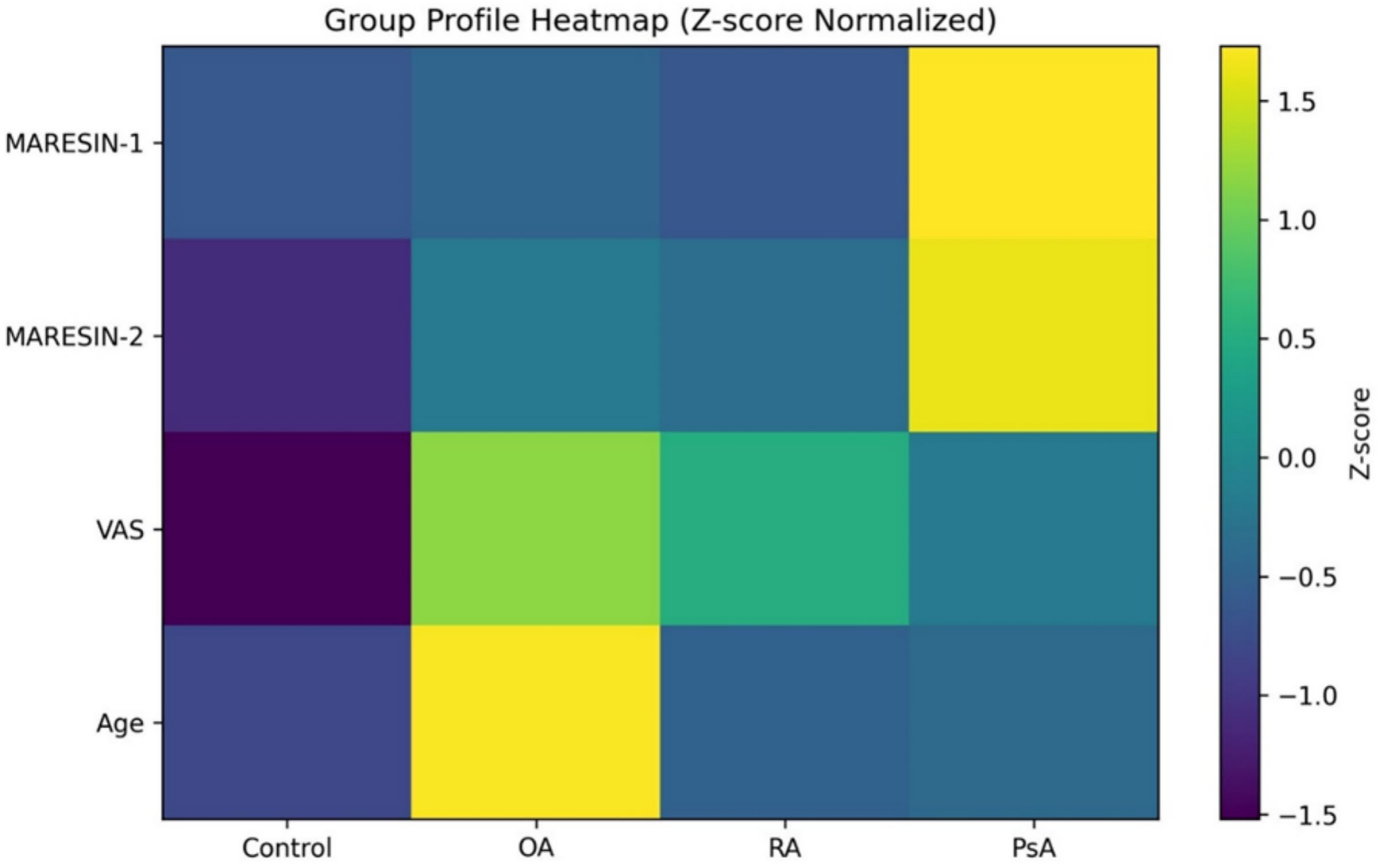
Z-score–normalized heatmap of group mean values for MaR-1, MaR-2, age, and VAS. MaR-1, maresin-1; MaR-2, maresin-2; OA, osteoarthritis; PsA, psoriatic arthritis; RA, rheumatoid arthritis; VAS, visual analogue scale.

When MaR-1 and MaR-2 were evaluated jointly in a combined model, discriminatory performance improved further, reaching an AUC of 0.864 (95% CI, 0.740–0.959; Table 3; Figure 5). In addition, group-wise profiles of MaR-1, MaR-2, age, and VAS scores are summarized using a z-score–normalized heatmap, illustrating their combined distribution patterns across PsA and RA groups (Figure 6). To account for the potential confounding effects of age and VAS score, a multivariable logistic regression analysis was performed. In this model, MaR-1 levels remained independently associated with PsA after adjustment for age, VAS score, and smoking status (adjusted OR = 1.52, 95% CI 1.18–1.96, *p* = 0.002). None of the other variables showed a statistically significant association (Table 4).

**Table 4:** Multivariable logistic regression analysis for differentiation of PsA from RA.

Variable	Adjusted OR	95% CI	*p*-value
MaR-1 (ng/mL)	1.52	1.18–1.96	0.002
Age (years)	1.01	0.98–1.04	0.412
VAS score	1.12	0.89–1.42	0.328
Smoking (yes/no)	1.34	0.56–3.21	0.504

MaR-1, maresin-1, VAS, visual analogue scale.

## 4 Discussion

In this prospective, observational, cross-sectional study, synovial fluid levels of MaR-1 in the knee varied significantly among the study groups, with markedly higher concentrations observed in patients with PsA compared with those with RA. The robust discriminatory performance of MaR-1 in differentiating PsA from RA suggests that this mediator may reflect active resolution processes within the synovial microenvironment. Maresins, members of the omega-3–derived specialized pro-resolving mediator (SPM) family, orchestrate the controlled termination of inflammation and promote tissue homeostasis, representing an active and tightly regulated biological process rather than simple suppression of inflammatory pathways (13–15). These findings gain additional significance when considered in the context of the distinct immunopathogenic mechanisms underlying PsA and RA, which likely influence the differential regulation and functional roles of pro-resolving lipid mediators in each disease.

In PsA, the relative dominance of the IL-23/IL-17 axis, entheseal-driven pathology, and innate immune mechanisms contrasts with the adaptive immunity–driven synovial pannus formation characteristic of RA, potentially resulting in distinct synovial lipid mediator landscapes between the two diseases (7, 8). Within this context, the elevated synovial MaR-1 levels observed in PsA may not simply reflect heightened inflammation but could represent an active, compensatory pro-resolving response within the joint aimed at terminating inflammation and restoring tissue homeostasis. However, due to the cross-sectional design of the study, it remains unclear whether the measured MaR-1 concentrations correspond to periods of active inflammatory flares, early resolution, or a broader adaptive response to chronic synovial inflammation. Importantly, these findings highlight MaR-1 as a central mediator in intra-articular resolution pathways, suggesting that its elevated presence may reflect the synovium’s intrinsic attempt to counterbalance ongoing immune activation rather than merely indicating disease severity.

Findings related to MaR-2 warrant a more cautious interpretation. In the present study, MaR-2 levels exhibited only borderline differences across groups and did not provide a degree of separation comparable to that observed for MaR-1. Previous experimental work has suggested that MaR-2 may exert context-dependent biological effects and can display analgesic and anti-inflammatory properties under certain conditions (33, 34). Accordingly, it is plausible that MaR-2 does not generate as robust a signal as MaR-1 for diagnostic discrimination in synovial fluid. Moreover, if the true effect size of MaR-2 is small, even a balanced design with 22 patients per group may have limited power to detect meaningful differences. From this perspective, the MaR-2 findings should be viewed not as definitively negative, but rather as secondary, hypothesis-generating results that require validation in larger cohorts and in analyses stratified by clinical activity and treatment status.

Assessment of lipid mediators in synovial fluid has predominantly been explored in the context of RA and OA, most often through lipidomic approaches (35–37). The characterization of lipid and lipid mediator profiles in RA synovial fluid using LC–MS/MS supports the notion that synovial fluid is a suitable biological matrix for the study of these mediators (10). In OA, growing evidence has highlighted the inflammatory spectrum of the disease, and targeted lipidomic studies in human knee OA have demonstrated that pro-resolving pathways can be activated within the joint environment (38, 39). Within this framework, integrating MaR-1 and MaR-2 patterns in OA with measures of synovitis burden, pain phenotype, and Kellgren–Lawrence stage may enhance the clinical relevance and interpretability of these biomarkers (38, 40).

The positive correlation observed between MaR-1 and MaR-2 in our study suggests that maresins may be co-regulated within the synovial microenvironment through shared biosynthetic pathways or similar cellular sources (33, 41). The stronger correlation seen in the PsA group further supports the hypothesis that resolution responses in PsA synovium may be characterized by a more coordinated lipid mediator profile. However, this interpretation requires confirmation through analyses stratified by disease activity and treatment status.

In future studies, the concurrent evaluation of MaR-1 and MaR-2 levels alongside synovial fluid cell counts and differentials, systemic inflammatory markers such as CRP and ESR, and validated clinical activity indices would help clarify whether maresins primarily reflect the magnitude of inflammatory burden or the capacity for inflammatory resolution (38, 40, 42).

Our ROC/AUC findings should be interpreted with caution in terms of clinical applicability. Although the discriminative performance of MaR-1 in distinguishing PsA from RA appears promising, cut-off values derived from a single-center cohort and based on cross-sectional measurements cannot be directly generalized to broader clinical settings. The development of a clinically meaningful biomarker would require validation across multiple centers, the use of standardized pre-analytical protocols, and rigorous control of potential confounders—such as age, VAS score, treatment exposure, and disease duration—through multivariable modeling. Where feasible, analytical confirmation using LC–MS/MS would further strengthen the validity of the findings (10, 38, 42).

Within this context, our study provides preliminary evidence in an area where clinical data comparing synovial MaR-1 and MaR-2 levels between PsA and RA remain limited. Without overextending claims of novelty, these results contribute to the existing literature and offer a rationale for larger, confirmatory investigations (38, 42–45).

Several limitations should be considered when interpreting the present findings. The single-center, cross-sectional design limits causal inference and precludes assessment of longitudinal changes in synovial MaR-1 and MaR-2 concentrations during disease progression or treatment response. Because measurements were obtained at a single time point, the dynamic relationship between maresin levels and inflammatory activity could not be evaluated; therefore, interpretation of these mediators as definitive “resolution markers” should be approached cautiously. In addition, inclusion was limited to patients with knee effusion, which may restrict the generalizability of the findings to other joint compartments and broader inflammatory arthropathy populations. Another important limitation is the absence of comprehensive disease activity and inflammatory assessments. Standardized indices such as DAS28, PASI, or DAPSA, as well as conventional inflammatory markers including CRP and ESR, were not systematically incorporated into the analyses, limiting the ability to establish clinically meaningful correlations between maresin levels and disease burden. Similarly, treatment-related variables—including conventional disease-modifying antirheumatic drugs (DMARDs), biologic therapies, and corticosteroid exposure—could not be fully evaluated and may have acted as potential confounders influencing synovial MaR concentrations. Nevertheless, the persistence of the association between MaR-1 and PsA after multivariable adjustment suggests that MaR-1 may reflect a distinct biological signal within the synovial microenvironment rather than merely mirroring demographic or clinical parameters such as age or pain severity.

The relatively modest sample size and lack of an *a priori* power calculation also warrant consideration. In particular, the weaker findings observed for MaR-2 may partly reflect insufficient statistical power and an increased likelihood of type II error. By contrast, the comparatively strong effect size and consistent discriminatory performance demonstrated by MaR-1 support the relative robustness of this observation despite the limited cohort size. Accordingly, these findings should be considered hypothesis-generating and require validation in larger, multicenter, longitudinal studies. From a clinical perspective, the most relevant implication of this study is the potential utility of MaR-1 as a supportive biomarker for differentiating PsA from RA, a distinction that carries important therapeutic implications in early disease stages. More broadly, the present findings support the emerging concept that specialized pro-resolving mediators derived from omega-3 fatty acids may participate in the immunobiology of inflammatory arthropathies and could represent future therapeutic targets. Further investigation into the effects of biologic therapies and omega-3–based interventions on maresin pathways may therefore provide valuable mechanistic and translational insights.

Lipid mediators are sensitive to pre-analytical variables such as sampling conditions, hemolysis, storage procedures, and freeze–thaw cycles, and some degree of measurement variability is therefore possible. Although ELISA was selected as an accessible and practical method, analytical validation using LC–MS/MS was not performed. Moreover, the handling of values at or near the lower and upper limits of detection may have influenced the distribution of extreme values. Finally, if the true effect size for MaR-2 is small, the present sample size may have been insufficient to detect statistically meaningful differences.

## 5 Conclusion

Synovial fluid concentrations of MaR-1 in the knee differed significantly among study groups, with markedly higher levels observed in patients with PsA compared with RA. The strong discriminatory capacity of MaR-1 in differentiating PsA from RA underscores the potential of pro-resolving lipid mediators as mechanistically informed biomarkers that reflect resolution biology within the joint microenvironment. In contrast, MaR-2 demonstrated less robust and consistent patterns, emphasizing the need for larger, well-powered studies with careful stratification by disease activity, therapeutic regimen, and clinical phenotype to fully elucidate its clinical relevance. These findings support the broader concept that targeting and monitoring resolution pathways may provide novel insights and opportunities for precision biomarker development in inflammatory arthropathies.

## Data Availability

The raw data supporting the conclusions of this article will be made available by the authors, without undue reservation.
